# The Periplasmic Domain of the Ion-Conducting Stator of Bacterial Flagella Regulates Force Generation

**DOI:** 10.3389/fmicb.2022.869187

**Published:** 2022-04-27

**Authors:** Michio Homma, Seiji Kojima

**Affiliations:** Division of Biological Science, Graduate School of Science, Nagoya University, Nagoya, Japan

**Keywords:** FliL, MotX, MotY, peptidoglycan, flagellar motor

## Abstract

The bacterial flagellar stator is a unique ion-conducting membrane protein complex composed of two kinds of proteins, the A subunit and the B subunit. The stator couples the ion-motive force across the membrane into rotational force. The stator becomes active only when it is incorporated into the flagellar motor. The periplasmic region of the B subunit positions the stator by using the peptidoglycan-binding (PGB) motif in its periplasmic C-terminal domain to attach to the cell wall. Functional studies based on the crystal structures of the C-terminal domain of the B subunit (MotB_*C*_ or PomB_*C*_) reveal that a dramatic conformational change in a characteristic α-helix allows the stator to conduct ions efficiently and bind to the PG layer. The plug and the following linker region between the transmembrane (TM) and PG-binding domains of the B subunit function in regulating the ion conductance. In *Vibrio* spp., the transmembrane protein FliL and the periplasmic MotX and MotY proteins also contribute to the motor function. In this review, we describe the functional and structural changes which the stator units undergo to regulate the activity of the stator to drive flagellar rotation.

## Introduction

**FIGURE 1:**
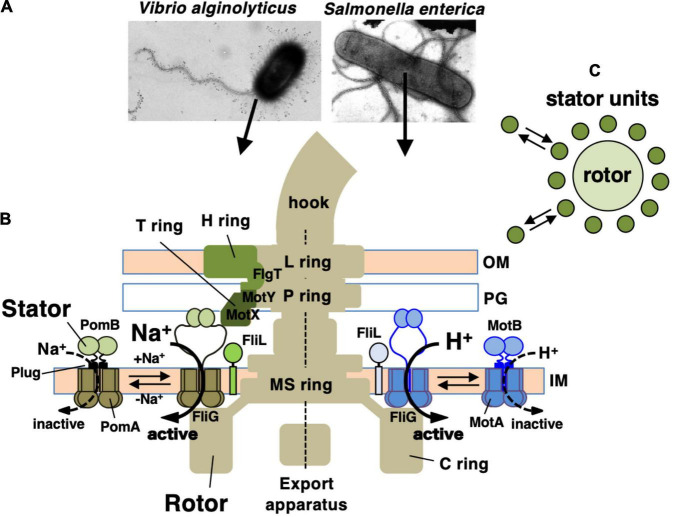
**(A)** Negatively stained bacteria were observed by electron microscopy in *Vibrio alginolyticus* (left side) and *Salmonella enterica* (right side). **(B)** Schematic drawing of the flagellum, which is a motility organelle that has a motor associated with the cell membrane. On the left is the sodium-driven polar flagellar motor of *V. alginolyticus*, and to the right shown the proton-driven flagellar motor of *S. enterica*. The *Vibrio* motor contains additional ring structures, the T ring and H ring, in addition to the L ring and P ring. The stator complex is composed of two integral membrane proteins, PomA and PomB, for the sodium-driven motor and MotA and MotB for the proton-driven motor. The stator is anchored to the PG layer around the rotor *via* the periplasmic domain of the B subunit. OM, outer membrane; IM, inner membrane; PG, peptidoglycan layer. FliL is located at the flagellar base close to the stator. **(C)** Schematic drawing of the dynamic assembly and disassembly of the stator units in the flagellar motor.

Many motile bacteria can swim in liquid or swarm on surfaces by rotating a structure called the flagellum (Beeby et al., 2020; Miyata et al., 2020). The flagellum consists of three parts: the long helical filament provides thrusts for the cell body, a rotary motor embedded in the cell surface, and a hook that connects the filament and the motor and serves as a universal joint. The flagellar motor rotates reversibly either clockwise (CW) or counterclockwise (CCW). Its energy source is an ion-motive force across the inner membrane: most of the bacteria, including *E. coli* and *Salmonella* spp., utilize H^+^, while *Vibrio* and some other species utilize Na^+^ (Figure 1A). The motor consists of a rotary part, the rotor (also called the flagellar basal body), which is composed of several rings and an axial rod that is connected to the hook; and the stator units, which are an energy-converting membrane protein complex (Terashima et al., 2008; Biquet-Bisquert et al., 2021; Hu et al., 2021). About a dozen stator units surround a single rotor. They are anchored at the peptidoglycan layer of the cell wall, and movement of the stator that is coupled to ion influx through the ion channel of the stator generates the rotational force (torque) (Figures 1B,C). Since the discovery of bacterial flagellar rotation in the 1970s (Berg and Anderson, 1973; Silverman and Simon, 1974), many researchers have been attracted to this rotary machine and studied the mechanism of its rotation, but still many details remain unknown.

To understand how the flagellar motor operates, the characterization of proteins in both the stator and the rotor is essential. Already in the 1990s, intensive genetic and biochemical analyses had identified most components of the motor. The locations of these proteins in the motor have been demonstrated by electron microscopic analyses of the isolated motor (Silverman and Simon, 1977; Macnab, 1992). In the 2000s, crystal structures of the rotor components were solved, and those structures were superimposed into the electron density maps of purified basal bodies obtained by cryo-electron microscopy (cryo-EM) (Kojima and Blair, 2004a; Minamino et al., 2008). Such analyses allow us to visualize the rotor structure in detail, and functional analyses to understand rotation from the rotor side, especially focused on rotational switching, have been conducted. In contrast, structural analysis of the stator has made little progress until recently. This is because the stator units dissociate from the flagellar basal body during isolation from the cell due to the weak interaction at the rotor–stator interface. However, in 2020, two groups reported high-resolution stator structures obtained by cryo-EM single-particle analyses (Deme et al., 2020; Santiveri et al., 2020). For details of the structural information, please see the review article in this issue of special topics on Biological Rotary Nanomotors.

Although the structural analysis of the stator units has lagged behind studies of the rotor, many genetic, functional, and biophysical characterizations of the stator units have been conducted (Minamino and Imada, 2015; Takekawa et al., 2020). Those efforts revealed unique features of the stator as an energy converter of the ion-motive force across the membrane. One of the notable properties is that the stator has two components: it has a static core, the B subunit, that is necessary to produce stable and constant torque, but it also has a dynamic periphery that allows it to deliver torque. The activity of the stator is regulated in response to environmental stimuli or load changes (Lele et al., 2013; Tipping et al., 2013; Antani et al., 2021). Here, we review the current state of knowledge about stator structure and function, focusing especially on its assembly-coupled mechanism of activation.

## The Stator Components

**FIGURE 2:**
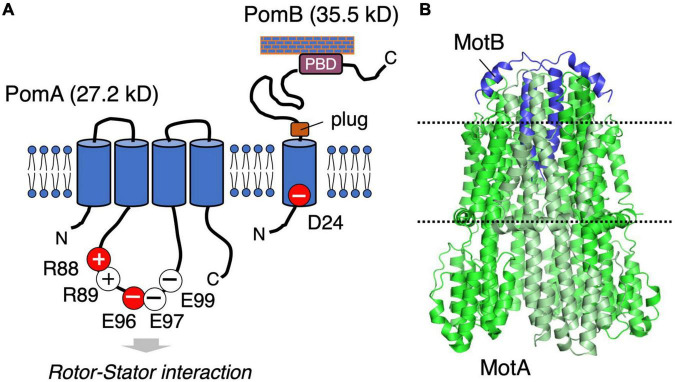
**(A)** Schematic of the stator structure. The stator of the sodium-driven motor is composed of the PomA and PomB proteins. PomA has important charged residues (R88, R89, E96, E97, and E99) in the cytoplasmic loop region. PomB has the essential charged residue (D24), which is the ion-binding site. **(B)** The structure of the flagellar stator (PDB: 6YKM) obtained by cryo-electron microscopy is shown in a ribbon diagram with MotA green and MotB purple. The dotted lines represent the predicted boundaries of the lipid bilayer.

The stator is composed of two membrane proteins: MotA and MotB for the proton-driven motor in *E. coli* and *Salmonella* (Silverman et al., 1976), PomA and PomB for the sodium-driven motor in *Vibrio* and *Shewanella* (Asai et al., 1997). MotA and PomA are orthologs, as are MotB and PomB, and both pairs share functionally critical residues and motifs at similar positions (Figure 2A). MotA/PomA have four transmembrane (TM) segments with a relatively large cytoplasmic loop between the second (TM2) and third (TM3) TM segments. This loop contains conserved charged residues that are important for electrostatic interactions with charged residues in the C-terminal domain of the rotor protein FliG. These electrostatic interactions are critically important for motor rotation (Yakushi et al., 2006; Takekawa et al., 2014). MotB/PomB have a single TM segment at their N-termini and a large periplasmic C-terminal region that is anchored to the peptidoglycan (PG) layer. The single TM of MotB/PomB contains a functionally critical aspartate residue (Asp32 in *E. coli* MotB, Asp24 of *Vibrio* PomB). This residue functions as a proton/sodium-ion-binding site in the stator and is essential for the stator function. Na^+^ binding at Asp24 of PomB was demonstrated using the Fourier Transform Infrared Spectroscopy (FTIR) (Sudo et al., 2009; Onoue et al., 2019). The periplasmic region of MotB/PomB contains a structurally conserved OmpA-like domain that is known to bind to the PG layer (Hizukuri et al., 2009). Mutations at a conserved PG-binding motif in this domain abolish motor rotation (Blair et al., 1991). Immediately C-terminal to the single TM of MotB/PomB is a characteristic amphipathic helix found in MotB family proteins (Hosking et al., 2006). Overproduction of the mutant stators with a deletion of this helix causes impairment of cell growth because of a massive proton- or sodium-ion flux through the stator ion channel. This segment is proposed to function as a “plug” of the stator channel to prevent premature ion flow when the stator is not incorporated into the motor (Hosking et al., 2006; Takekawa et al., 2013). In the 2000s, biochemical analyses and comprehensive disulfide crosslinking between the TM segments revealed the multimeric structure of a stator complex in which two MotB molecules were proposed to be surrounded by four MotA molecules (Braun and Blair, 2001; Blair, 2003; Braun et al., 2004; Kojima and Blair, 2004b). This model was updated by the high-resolution cryo-EM structure of the MotA/MotB stators reported in 2020: five, not four, MotA molecules form a ring that surrounds a central MotB dimer (Deme et al., 2020; Santiveri et al., 2020; Figure 2B). The structure inspired a novel model for torque generation in which a pentameric MotA ring rotates around the MotB dimer in response to ion influx; this rotation drives the rotor (Hu et al., 2021). In this structure, the C-terminal periplasmic region following the plug segment of the MotB was not visible.

## Dynamic Properties of the Stator Units

Although the details of the stator structure have only recently been elucidated, biophysical studies provided many insights into the properties of a single stator unit. Berg and coworkers developed the “resurrection” experiments in which the plasmid-borne stator restores rotation to the motor of a tethered stator-defective strain (Block and Berg, 1984). They found the stepwise recovery of rotation speed with the same increment in speed for each step. Similarly, a stepwise decrease in the rotational speed of alkalophilic *Bacillus* sodium-driven motor was seen after UV-induced irreversible inhibition of the stator (Muramoto et al., 1994). These results indicate that each stator unit is incorporated into the motor and functions independently. Later, resurrection experiments were conducted with a low viscous load on the flagellum using a bead attached to the flagellar stub (Ryu et al., 2000; Sowa et al., 2005). It was found that around eleven stator units can be incorporated into the fully functional motor (Reid et al., 2006) and that a single motor rotates with 26 steps per revolution, a number consistent with the periodicity of the ring of the FliG protein in the rotor (Sowa et al., 2005).

Motor rotation, observed at a high-load condition using tethered cells or at low-load using laser-dark field microscopy or a bead-flagellar stub system, appears to be quite stable (Kudo et al., 1990; Ryu et al., 2000). This stability suggests that stator units are static and, once incorporated into the motor, are stably anchored. However, another property of the stator was revealed by the observation of a single stator unit *in vivo*, using green fluorescent protein (GFP, fusion to MotB) (Leake et al., 2006). This study showed that the stator units in a functional motor can be exchanged rapidly with a turnover rate of only ∼0.5 min. Similar dynamic properties were observed in the Na^+^-driven PomA/PomB stator in *Vibrio* and *Shewanella* motors (Fukuoka et al., 2009; Paulick et al., 2009). The stator dissociates from and associates with the rotor in response to a changing Na^+^ concentration. These findings have changed the image of the stator as being static: the stator has dynamic properties that respond to the environmental conditions. Indeed, evidence has accumulated to show that the stator works as a mechano-sensor: the stator number changes in response to the load on the motor (Armitage and Berry, 2020). As the environmental load increases, the number of stator units around the rotor increases. Under a high-load condition, the tension applied on the binding interface between the PG layer and MotB promotes conformational changes that further expose additional binding residues in MotB to tighten the non-covalent binding between MotB and the PG layer to increase the lifetime of the stator around the rotor. Such a “catch-bond” mechanism equipped in MotB/PomB leads to an increase in the number of stators that enables the cells to rotate the motor in high viscous load (Nord et al., 2017). New insights were reported recently showing the involvement of the rotor side for mechanosensing in the flagellar motor (Antani et al., 2021).

**FIGURE 3:**
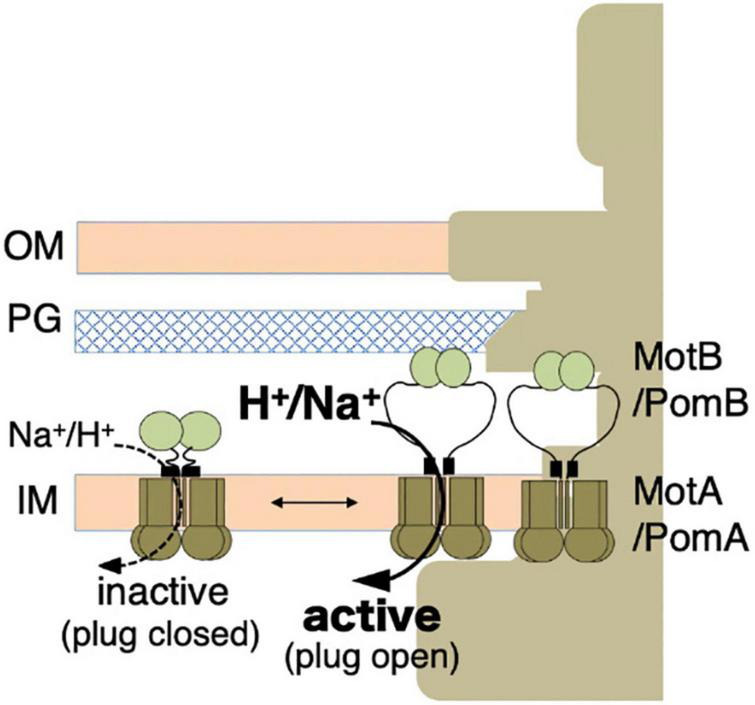
Stator activation by the two-state model. The stator, composed of MotA or PomA and MotB or PomB, is activated by the coupling ions. The plug region is open in the active state. OM, outer membrane; IM, inner membrane; PG, peptidoglycan layer.

The overproduction of the full-length stator complex does not affect cell growth, suggesting that the channels of unincorporated stator units are closed (Wilson and Macnab, 1988; Stolz and Berg, 1991). Rapidly diffusing intact stator units have been observed in the membrane, indicating that unincorporated stators are not anchored to the PG layer (Fukuoka et al., 2007, 2009). Considering the dynamic properties described above, a stator should have at least two distinct states: (i) an unincorporated inactive state with the ion channel closed and no attachment to the PG layer; (ii) an incorporated active state with the ion channel open and MotB anchored to the PG layer (Figure 3).

## Assembly-Coupled Activation Mechanism of the MotA/MotB Stator

**FIGURE 4:**
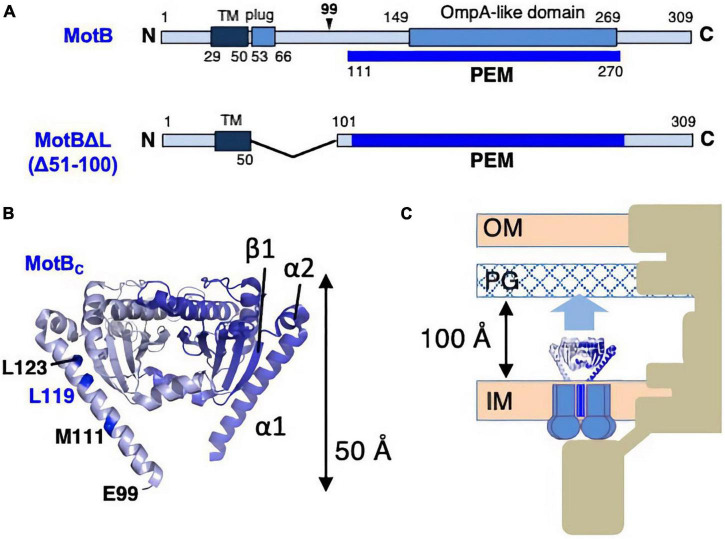
**(A)** Primary structures of *Salmonella enterica* MotB; the PEMs for MotB are shown by the blue lines below each primary structure. **(B)** Dimer structures of the periplasmic fragments (MotB_*C*_) of *Salmonella* MotB (PDB: 2ZVY). **(C)** Schematic drawing of the compact conformation of the stator complex composed of MotA and MotBΔL. OM, outer membrane; IM, inner membrane; PG, peptidoglycan layer.

The inactive, unincorporated, and active incorporated forms of the stator may have evolved to prevent wasting the energy by unincorporated stators. It requires that there must be a regulatory mechanism by which the stator is activated only when it is incorporated into the motor. How does such activation occur? It is plausible that the key conformational change is induced in the periplasmic region of MotB/PomB, where the binding to the PG layer must occur. To test this idea, the crystal structure of the periplasmic region of *Salmonella* MotB was determined (Kojima et al., 2009). Deletion studies of MotB identified the periplasmic region of MotB as essential for motility, which we call PEM (residues 111–270) (Muramoto and Macnab, 1998; Figure 4A).

Various fragments containing the entire PEM were constructed, and the crystal structure was determined for one of those fragments, MotB_*C*2_ (residues 99–276), which was crystallized (Figure 4B). MotB_*C*2_ forms a homodimer and appears in an unexpectedly compact conformation (Kojima et al., 2009). It has a single domain with considerable similarity to known OmpA-like domain structures. This domain is connected to a long N-terminal long α-helix (α1), which is followed by a short helix (α2) and a β-strand (β1). MotB/PomB proteins from various bacterial species do not show sequence similarity at the N-terminus of the PEM, but secondary structural predictions suggest that α1, α2, and β1 are common structural features. A MotB protein deleted for residues 51–100 (named MotBΔL), in which the TM segment is directly connected to the crystallized region of MotB_*C*2_, is known to function (Muramoto and Macnab, 1998), so the MotBΔL stator must be anchored at the PG layer. However, the MotB_*C*2_ dimer is only 50 Å tall and is, therefore, too short to reach the PG layer: the distance between the PG layer and the surface of the hydrophobic core layer of the cytoplasmic membrane is about 100 Å (Figure 4C). Therefore, a large conformational change must be induced in the PEM when the stator is incorporated into the motor. Since the OmpA-like domain is structurally well conserved, such structural change must occur in the N-terminal PEM (α1, α2, β1).

What kind of conformational changes are likely to occur? If the β1 detaches from the OmpA-like domain and extends collinearly with the α1 and α2 helices, the entire PEM of MotBΔL is now long enough to reach the PG layer. This model is supported by structure-guided mutagenesis in the N-terminal PEM, which shows that a Pro or Glu replacement at Leu119 in α1 of MotBΔL affects cell growth when co-overproduced with MotA (Kojima et al., 2009). The measurement of cytoplasmic pH revealed that this growth inhibition was caused by high proton conduction by this mutant stator (Morimoto et al., 2010a). These results suggest that the L119P (or L119E) substitution alters the stator structure in a way that mimics the active state. Inconsistent with this idea that low-level expression of this mutant still allows motility although the MotA/MotBΔL stator requires overexpression for function. The MotA/MotBΔL (L119P/E) stator exhibits more favorable conformation than MotA/MotBΔL stator for efficient incorporation into the motor.

MotB is believed to bind to the PG layer *via* the conserved PG-binding motif in the structurally conserved OmpA-like domain; mutations targeting the PG-binding motif abolish cell motility (Blair et al., 1991) and localization of the stator around the rotor (Fukuoka et al., 2009). However, little direct evidence for this interaction has been reported. Only a very weak interaction between the isolated PG layer and the periplasmic fragment of *Helicobacter* MotB was observed (Roujeinikova, 2008; Andrews et al., 2017). If the L119P mutation alters the conformation of PEM so that it mimics the conformation in the active state of the stator, then this mutant should bind to the PG layer. To test this idea, the PG-binding activity of the MotB_*C*2_-L119P fragment was investigated by the co-sedimentation assay (Kojima et al., 2018). Although wild-type MotB_*C*2_ did not co-precipitate with the isolated PG and remained in the supernatant, most of the MotB_*C*2_-L119P co-precipitated with PG. Therefore, the L119P replacement in MotB_*C*2_ changes its conformation such that it can bind to the PG layer.

What kind of conformational change is induced by the L119P substitution? First, solution NMR analysis of selectively labeled MotB_*C*2_ with (α-)^15^N-lysine was conducted to investigate the structure in the solution (Kojima et al., 2018). The NMR data revealed that structural changes caused by the L119P mutation were localized in helix α1, not in the OmpA-like domain. Next, the crystal structure of MotB_*C*2_-L119P protein was solved (Kojima et al., 2018). Consistent with the NMR results, the crystal structure of the MotB_*C*2_-L119P dimer was almost identical to that of MotB_*C*2_ except the helix α1 was disordered and not visible in the structure. No significant structural changes were found in the putative PG-binding residues in the OmpA-like domain. Since the MotB_*C*2_-L119P fragment showed the PG-binding property, the conformational change that occurs in helix α1 unmasks/exposes additional residues in the OmpA-like domain required for PG-binding. The L119P substitution seems to induce a structural change that converts helix α1 into an extended open conformation. We propose that this rearrangement is responsible for stator activation both for PG-binding and proton conductivity.

To test whether similar conformational changes occur *in vivo*, crosslinking assays were conducted in intact cells. The MotA/MotBΔL stator with double-cysteine replacements in the PEM region, one in helix α1 (I127C) and the other in the PG-binding core (L140C), was constructed (Kojima et al., 2018). Cells expressing this mutant stator were only slightly motile both in semisolid agar and in the liquid, but the addition of the reducing agent dithiothreitol (DTT) dramatically improved motility, suggesting that the disulfide bridge formation between I127C and L140C residues reversibly inhibits motility. This *in vivo* assay provided evidence for a conformational change in the entirety of helix α1 during stator incorporation into the motor.

**FIGURE 5:**
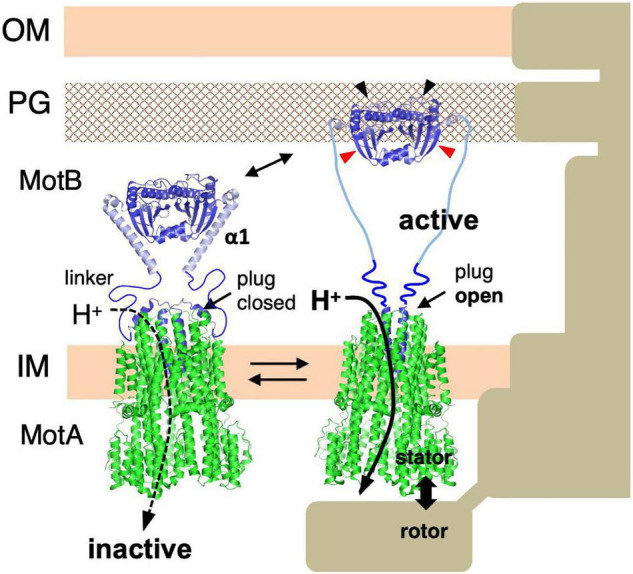
Molecular model of stator activation. In the MotB protein, the crystallized MotB_*C*_ region (PDB: 2ZVY) was directly connected to the transmembrane segment by way of the linker region and plug regions. A conformational change must be induced in MotB to enable it to reach the PG layer (black arrows). The structures of the flagellar stator (PDB: 6YKM) and the periplasmic fragments (MotB_*C*_) of *Salmonella* MotB (PDB: 2ZVY) shown by a ribbon diagram are connected with a linker (left image). The structures of the unplugged stator (PDB: 6YKP) and the periplasmic fragments (MotB_*C*_) of *Salmonella* MotB L119P mutant (PDB: 5Y40) are shown by a ribbon diagram are connected by the linker and the disordered helix α1 region (right image). OM, outer membrane; IM, inner membrane; PG, peptidoglycan layer.

Based on these results, a model for the assembly-coupled MotA/MotB stator activation was proposed: (i) Stator units diffuse in the cell membrane in an inactive state. (ii) When they contact the rotor, helix α1 of MotB changes from a compact form into an extended open conformation. (iii) This conformational change pulls the plug to allow efficient proton translocation through the ion channel in the stator. (iv) At the same time, the conformational change extends the PG-binding domain of MotB to the PG layer and exposes/unmasks the region essential for PG-binding. The stator can now bind to the cell wall and generate/transmit the torque to the motor (Figure 5).

## Activation Mechanism of the Na^+^-Driven *Vibrio* Stator

**FIGURE 6:**
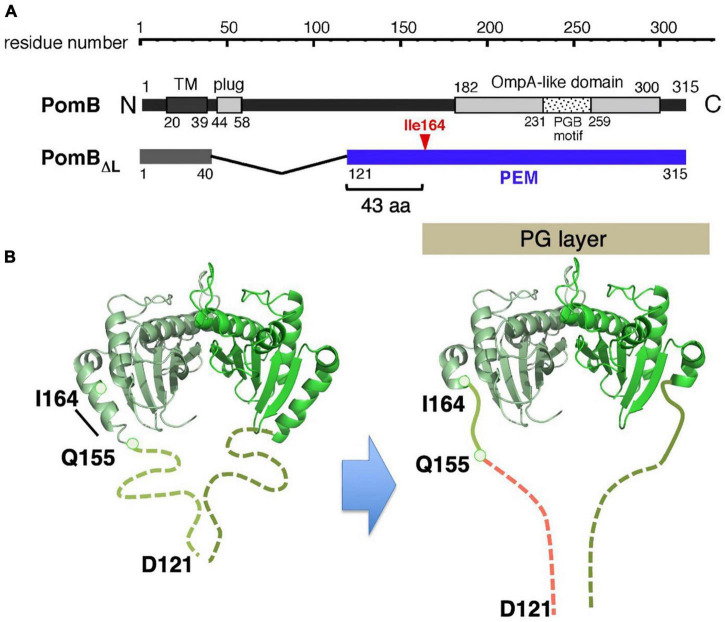
**(A)** Primary structure of *Vibrio alginolyticus* PomB. The PEM for MotB is shown by the blue line in the in-frame deletion mutant (PomB_Δ*L*_). **(B)** A model of the conformational change in PomB coupled to the stator assembly. The periplasmic fragments (PomB_*C*_) of *Vibrio* PomB (PDB: 3WPW) shown by the ribbon diagram are connected with a linker (dotted lines) (left). The N-terminal two-thirds of helix α1 change conformation to an extended form that is able to anchor to the PG layer (right).

All the stators, regardless of the bacterial species of coupling ions they use (H^+^ or Na^+^), could use the same mechanism for their assembly-coupled activation. To address this point, the conformational change in the sodium-driven stator protein PomB of *Vibrio alginolyticus* was investigated. Deletion studies identified the PEM region of PomB as consisting of residues 121–315 (Li et al., 2011), and crystal structures of PomB fragments encoding PEM (PomB_*C*4_, residues 121–315; PomB_*C*5_, residues 135–315) were solved (Zhu et al., 2014; Figure 6A). The structures of PomB_*C*4_ and PomB_*C*5_ are identical, as the residues before 154 and after 305 are disordered in both structures. The solved structure we describe here as PomB_*C*_, is quite similar to that of MotB_*C*2_, forming a homodimer consisting of a single OmpA-like domain with a characteristic N-terminal helix (α1) (Figure 6B). The major difference between MotB_*C*_ and PomB_*C*_ is in the N-terminal PEM, including helix α1. In PomB_*C*_, residues 121–154 are disordered in the crystal and α1 is 10 residues shorter than in MotB_*C*_. It should be noted that in the *Vibrio* motor, stator units must interact not only with the PG layer but also with the T ring in the basal body (Terashima et al., 2006). Even though PomB_*C*_ has a more flexible region in the PEM (the smallest functional PomB mutant named PomBΔL, is deleted for residues 41–120), the compact PomB_*C*_ dimer still appears to be too small to reach the PG layer and T ring. Therefore, it is likely that PomB changes the conformation of its N-terminal PEM upon incorporation into a motor, as proposed in MotB.

To test this possibility, an *in vivo* disulfide crosslinking approach, similarly conducted for MotB, was employed (Zhu et al., 2014). It was expected that an intramolecular disulfide crosslink between helix α1 and the PG-binding core would impair any major conformational change and abolish motility. Indeed, the crosslink between M157C (in α1) and I186C (in the PG-binding core) abolished motility and the reduction of this crosslink by DTT restored motility. However, the slightly more C-terminal I164C-V179C crosslink still allows motility. This result is inconsistent with the previous model that strand β1 in PomB_*C*_ (or MotB_*C*_) extends to release the PG-binding core for anchoring the stator, but it is consistent with the updated model that the N-terminal PEM, including helix α1, is responsible for the conformational change upon stator activation. In the case of *Vibrio* PomB, only the N-terminal two-thirds of α1 changes its conformation. This partial rearrangement of the α1 helix, along with a conformational change in the adjacent N-terminal disordered region (residues 121–154), would allow PomBΔL to reach the PG layer.

We conclude that at least some H^+^ and Na^+^ stator units share the common mechanism for assembly-coupled activation. It should be noted that in the original paper that reported the PomA/PomB activation, a two-step conformational change was proposed, in which the first conformational change occurs in the disordered region in the N-terminal PEM to interact with the T ring, and the second conformational change occurs in the N-terminal two-thirds of α1 to reach the PG layer (Zhu et al., 2014). This model was based on the observation that the M157C-I186C crosslink in full-length PomB abolished motility but still allowed localization of PomAB to the motor. Subsequent cryo-electron tomography revealed a globular density at the periphery of the T ring (Zhu et al., 2020). Because the crystal structure of PomB_*C*_ dimer fits well to this density, which is not observed in the motor of Δ*pomAB* strain, it was suggested that the *Vibrio* PomA/PomB stator is anchored to the T ring, instead of the PG layer. Therefore, the N-terminal PEM seems not to be the binding site for the T ring. The two-step conformational change model (Figure 3) in PomB for stator activation must be tested in the light of the PomB_*C*_ interaction with the PG layer or with MotX, the component located at the tip of the T ring.

## Pulling the Plug to Allow Ion Flow Through the Stator

It has been proposed that the interaction of the stator with the rotor induces the opening of the ion channel (Kojima et al., 2011). The plug segment, a characteristic amphipathic helix located immediately at the C-terminal of a single TM segment of the B subunit, is proposed to regulate the ion-conducting activity of the stator, by “plugging” the channel (Hosking et al., 2006; Takekawa et al., 2013). How unplugging occurs during stator activation is not yet known. However, the high-resolution structures of the MotA/MotB stators included the plug helix and combined with a functional analysis revealed its novel role. The structure shows that the plug helix lies between the MotA subunits in the pentameric MotA ring, with three MotA subunits on one side and two on the other (Deme et al., 2020; Santiveri et al., 2020). The interaction of the plug with the MotA ring interferes with the ion flux, presumably by affecting the structure of the ion pathway, and the disruption of this interaction allows efficient ion flux through the stator channel. As discussed above, stator activation would induce movement of, or a conformational change in, the plug helix to open the channel.

**FIGURE 7:**
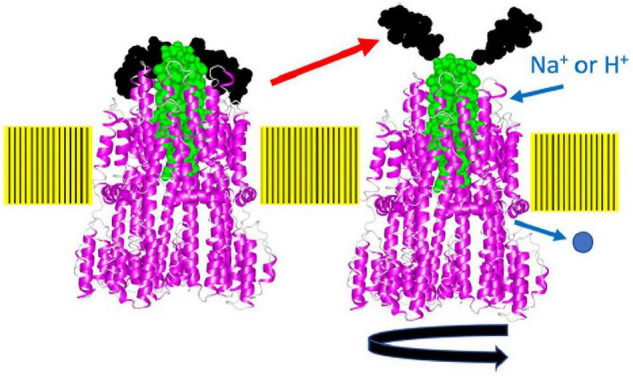
A model of how the plug may block stator rotation and stator activation. When the cytoplasmic region of MotA or PomA interacts with FliG, the plug region of PomB or MotB, which seems to function as a spanner or stopper, is released from the periplasmic loop regions (the putative right structure). The pentamer of the A subunit rotates around the B subunit driven by ion flow. The structure of the flagellar stator (PDB: 6YKM) which is stopped state (the left structure), is shown by a ribbon diagram, with MotA in magenta and MotB in green. The spanner region is shown in black in a space-filling model. This figure is taken from our previous paper (Homma et al., 2021).

Recently, the site-specific photograph-crosslinking between the PomA periplasmic loop and the plug helix of PomB was conducted. The results are consistent with the MotA/MotB structure (Homma et al., 2021). A disulfide crosslink between PomA (M169C in the large periplasmic loop) and PomB (I50C in the plug) reversibly inhibits motility. The structure of the stator complex implies that the MotA ring rotates around the MotB dimer in response to ion flux. The inhibition of motility suggests that the crosslink between the plug and MotA ring interferes with ion influx through the stator by physically blocking the rotation of the stator ring like a spanner or a stopper (Figure 7). This model is supported by the observation that a plug deletion in PomB weakens the PomA–PomB interaction and results in their dissociation from each other during the purification (Nishikino et al., 2020). It should be noted that the plug deletion of the plug in MotB of *Campylobacter jejuni* did not weaken the MotA–MotB interaction, and the plug-deleted stator complex could be purified and used for the structural analysis (Santiveri et al., 2020).

## Components That Stabilize Active Stator

**FIGURE 8:**
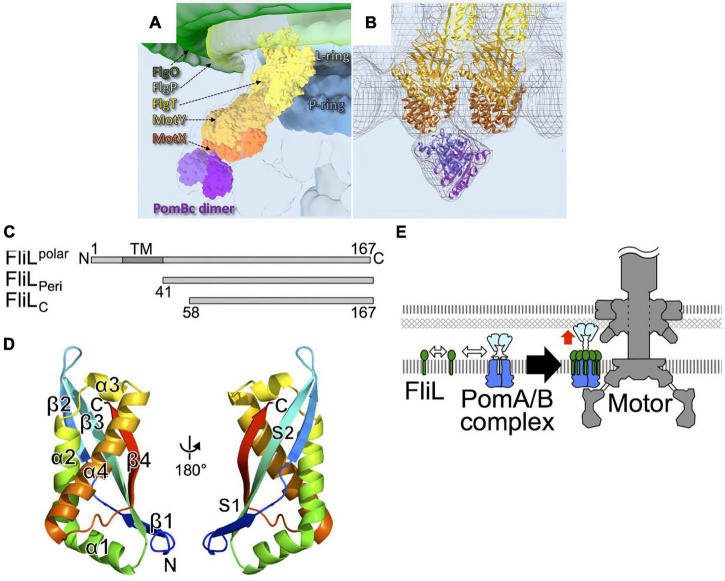
**(A)** A 3D surface rendering of the part of the *Vibrio* flagellar basal body including the L ring and the P ring in addition to the O ring, the H ring, and the T ring. The location of the H ring is inferred based on our previous results. FlgO is labeled in deep green and located at the distal area of the H ring, and FlgP is labeled in light green and located in the middle of the H ring. FlgT might be located outside of the P/L rings and on the top of the T ring. **(B)** Superposition of the cryo-ET density map (mesh) and the atomic models of FlgT, MotY, MotX, and PomB, as previously shown (Zhu et al., 2020). The models are shown as ribbon diagrams and colored as in **(A)**. **(C)** Primary structure of *Vibrio alginolyticus* FliL and the truncations, FliL_*Peri*_, and FliL_*C*_. **(D)** The structure of FliL_*C*_ (PDB: 6AHP) is shown as a ribbon diagram colored like a rainbow from the N-terminus (blue) to the C-terminus (red). **(E)** A model for the role of FliL in stator assembly. The white two-way arrows indicate the protein-protein interactions of the stator with FliL, and the red arrow indicates the activation of the stator. Figures are taken from our previous papers: Zhu et al. (2020) for **(A,B)**, Takekawa et al. (2019) for **(C–E)**.

As described above, stator units exhibit dynamic properties. They undergo rapid turnover, which involves frequent association and dissociation with the motor. However, the motor rotates quite stably, indicating that incorporated stators can be stabilized in the motor. The sodium-driven *Vibrio* polar flagellar motor, as discussed in the previous section, rotates remarkably fast, up to ca. 1,700 Hz, as compared to the proton-driven motors of *E. coli* and *Salmonella* which rotate up to ca. 300 Hz (Kudo et al., 1990; Gabel and Berg, 2003). The basal body of the *Vibrio* polar flagellum has unique ring structures, the T ring and H ring (Terashima et al., 2006, 2013). These extra rings are thought to allow the motor to perform high-speed rotation. The T ring is located beneath the H ring and is required for the assembly of PomA/PomB around the rotor. It is presumably also important for the stabilization of the active stator in the motor. The T ring is composed of MotX and MotY, and the loss of either abolishes motor rotation (Terashima et al., 2006). MotY has a two-domain structure, with a unique N-terminal fold that is responsible for interaction with MotX and the basal body, and with a C-terminal OmpA-like domain that stabilizes stator association by binding to the PG layer. The structure of MotX has not yet been solved because its precipitation hampers its purification. MotX has a characteristic SEL1 motif, a repeat of α helices that is involved in protein-protein interaction, and an *in silico* structure has been predicted (Zhu et al., 2020). Cryo-electron tomographic analysis of *Vibrio* polar flagellar motor shows that this predicted structure of MotX fits well into the T ring density if MotX and MotY form a 1:1 heterodimer, with MotX positioned at the tip of the T ring (Figures 8A,B). Whatever the actual structure may be, the interaction between MotX in the T ring and PomB in the stator stabilizes the active stator units incorporated around the rotor.

FliL is another component that stabilizes the active stator. When FliL is not present, motility is impaired when the cells swim or swarm in a highly viscous environment. Under such conditions, the motor must turn against the large friction imposed by a high load, and the stator units must associate tightly with the rotor to generate maximal torque. A catch-bond mechanism in the stator B subunit would contribute to this mechano-sensing reaction. However, an additional factor is required under high-load conditions, presumably to stabilize the active stator units around the rotor. FliL is a small protein with a single transmembrane segment. Most of it is located in the periplasm (Figure 8C). It is essential for the swimming motility of some species, including *Caulobacter crescentus* (Jenal et al., 1994), *Rhodobacter sphaeroides* (Suaste-Olmos et al., 2010), and for the surface swarming of *E coli* and *Salmonella* (Attmannspacher et al., 2008). It should be noted that the torque generated by the *E. coli* strain lacking FliL is reported to be similar to that in the wild-type strain at high-load conditions (Chawla et al., 2017), raising a question about FliL involvement in mechanosensing by the motor. FliL is encoded in an operon containing other flagellar genes, and it associates with the flagellar basal body (Partridge et al., 2015). Because it is not essential for swimming motility in *Salmonella* and *E. coli*, its function has not been intensively studied although it was identified a while ago (Raha et al., 1994). Recent work has revealed the diverse functions of FliL. Cryo-electron tomography revealed that, in *Borrelia burgdorferi* motor, FliL is located close to the rotor and stator (Motaleb et al., 2011). FliL interacts with basal body proteins such as FliF of the MS-ring protein and the FlgT protein associated with the H ring. Most importantly for this discussion, it also interacts with the periplasmic region of the B subunit in the stator. FliL-stator interaction is important for the localization of FliL at the motor (Lin et al., 2018). It has been shown that *Vibrio alginolyticus* polar FliL forms an oligomer (ca. 150 kDa) in a detergent (Kumar et al., 2017). The crystal structure of the periplasmic region of FliL (FliL_*P*_) shows remarkable structural similarity to the mammalian stomatin/prohibitin/flotillin/HflK/C (SPFH) domain of stomatin (Figure 8D). The SPFH domain is conserved in membrane-associated proteins of eukaryotes, where it is known to interact with various ion channels and transporters to modulate their activities (Takekawa et al., 2019). Furthermore, proteins with the SPFH domain are often involved in the mechanosensing of sensory neurons, raising the possibility that FliL is also involved in mechanosensing by the flagellum.

The structure of FliL_*P*_ and functional studies suggest that FliL forms a multimer in the periplasm, possibly a decameric ring, which surrounds a single stator unit. One model proposes that 10 molecules of FliL surround a single PomA/PomB stator unit. The FliL–PomB interaction in the periplasm stabilizes the active conformation of a stator whose ion channel is open and is anchored to the PG layer or T ring (Takekawa et al., 2019; Guo et al., 2022; Tachiyama et al., 2022; Figure 8E). FliL may also assist in the catch-bond function of the PG-binding core of PomB. Although the high-resolution structure provided insight, it is still not clear how FliL senses the environmental load and modulates stator function. Further studies will be needed to test how prevalent it is to have a FliL ring around a stator unit and to determine the molecular details of the stator–FliL interaction.

## Remaining Question: What Is the Trigger?

Structure-based functional analyses revealed the conformational change in the PEM region of the stator B subunit that alters the stator structure to the active conformation. What triggers this conformational change? The short answer is, we do not know. Because the stator unit is activated only when it is incorporated into the motor. It is most likely that rotor–stator contacts provide the signal for stator activation (Fukuoka et al., 2009). Genetic studies have provided information about the rotor–stator interaction in the cytoplasm, which occurs between conserved charged residues in the loop between TM2 and TM3 of the A subunit of the stator, and the C-terminal domain of the rotor protein FliG (FliG_*C*_) (Kojima et al., 2011). Genetic suppression and synergistic effects between these charged residues indicate that electrostatic interactions at the rotor–stator interface are critical for motor rotation (Zhou et al., 1998; Takekawa et al., 2014). Therefore, a plausible scenario is that once a diffusing stator unit in the membrane encounters a rotor, electrostatic interactions at the rotor–stator interface trigger a signal that is transmitted to the periplasmic side of the stator to induce the conformational change of PEM in the B subunit.

Although this hypothesis is attractive, more experimental evidence is needed to prove the model. Some evidence has been reported to show that the proper interactions between rotor and stator are required for stator incorporation/assembly into the motor. In the *Salmonella* motor, the investigation of stator localization using a C-terminally GFP-tagged MotA revealed that electrostatic interaction between MotA (Arg90)-FliG (Asp289) was critical for proper positioning of the stators around the rotor, whereas that of MotA (Glu98)-FliG (Arg281) is more important for torque generation (Morimoto et al., 2010b,^2013^). In the *Vibrio* motor, mutations targeting the cytoplasmic region of the A subunit or FliG_*C*_ abolish the stator localization around the rotor (Kojima et al., 2011; Takekawa et al., 2012). These results support the idea that the rotor–stator contact acts as a trigger. A possible intermediate state in the association process was detected in the *Vibrio* PomA/PomB stator in the presence of coupling ion Na^+^, by site-specific labeling experiments (Mino et al., 2019). This state presumably represents the conformation before the activation, as it was observed in a mutant stator missing a critical Na^+^ binding site in TM of PomB. In the MotA/MotB stator, such an intermediate state has not been reported yet.

Recently, the direct physical interaction between the rotor and stator was demonstrated in the *E. coli* hybrid motor by using the Na^+^-driven PomA/PotB chimeric stator and the site-specific photograph- and disulfide crosslinking *in vivo* (Terashima et al., 2021). PotB is the chimera consisting of an N-terminal PomB fusing to the periplasmic C-terminal MotB (Asai et al., 2003). In this study, a photograph-reactive amino acid derivative (*p*-benzoyl-_*L*_-phenylalanine, pBPA) was used to replace a series of residues at the rotor–stator interface. UV-irradiation induces a covalent photograph-crosslink with nearby residues. This approach allowed the detection of weak or transient interactions at the rotor–stator interface. The results show that the region of PomA containing conserved charged residues indeed interacts with the region containing charged residues in FliG_*C*_, and some specific interaction pairs were discovered. Because the positively or negatively charged residues are located next to each other in the recently solved high-resolution structure of MotA, the rotation gear model was proposed, in which the stator A subunit ring rotates against the rotor ring by alternate electrostatic repulsion and attraction in coupling with ion flux through the rotor.

A cryo-electron tomography analysis of the *Borrelia* motor *in situ* revealed the structural remodeling that accompanies rotational switching (Chang et al., 2020). It was proposed that a stator ring changes its contact sites to the rotor depending on the rotational directions (counterclockwise or clockwise). Because the crosslinking studies described above used a strain whose motor rotates in both directions, the contact residues at the rotor–stator interface may be the same regardless of the rotational direction. Therefore, stator activation may be induced when the motor is in either direction of rotation *via* electrostatic interactions of the same charged residues. However, further analyses are required to clarify the activation mechanism.

This still leaves an open question of how the signal from the rotor–stator contact is transmitted to the PEM region of the B subunit, which is distant from the contact interface. In the current model, we propose that conformational changes in helix α1 of the N-terminal PEM of MotB simultaneously open the plug and expose PG-binding determinants (Kojima et al., 2018). The cytoplasmic signal may induce unplugging. This movement of the plug segment could induce the rearrangement of helix α1. To understand the series of conformational rearrangements in the stator that occur during assembly-coupled activation, we need the whole structure of the part of the periplasmic domain of the B subunit that is currently invisible. However, having most of the structure of the stator allows us to conduct structure-based functional analysis. We also have a variety of methods to analyze the behavior of each stator unit. We can observe and visualize the single stator unit *in vivo* by using state-of-art light or electron microscopy. High-speed atomic force microscopy would be suitable to characterize *in vitro* physicochemical properties of the stator *in vitro*. We should soon be able to understand the assembly, activation, and function of the stator units, which are unique energy-converting complex in the bacterial cell membrane.

## Author Contributions

Both authors listed have made a substantial, direct, and intellectual contribution to the work, and approved it for publication.

## Conflict of Interest

The authors declare that the research was conducted in the absence of any commercial or financial relationships that could be construed as a potential conflict of interest.

## Publisher’s Note

All claims expressed in this article are solely those of the authors and do not necessarily represent those of their affiliated organizations, or those of the publisher, the editors and the reviewers. Any product that may be evaluated in this article, or claim that may be made by its manufacturer, is not guaranteed or endorsed by the publisher.
